# Numerical model of optical coherence tomographic vibrography imaging to estimate corneal biomechanical properties

**DOI:** 10.1098/rsif.2014.0920

**Published:** 2014-12-06

**Authors:** Sabine Kling, Imran B. Akca, Ernest W. Chang, Giuliano Scarcelli, Nandor Bekesi, Seok-Hyun Yun, Susana Marcos

**Affiliations:** 1 Instituto de Óptica, Consejo Superior de Investigaciónes Cientificas, Madrid, Spain; 2 Wellman Center for Photomedicine and Harvard Medical School, Massachusetts General Hospital, Boston, MA, USA

**Keywords:** non-contact, corneal biomechanical properties, vibrography, corneal natural frequencies

## Abstract

Most techniques measuring corneal biomechanics *in vivo* are biased by side factors. We demonstrate the ability of optical coherence tomographic (OCT) vibrography to determine corneal material parameters, while reducing current prevalent restrictions of other techniques (such as intraocular pressure (IOP) and thickness dependency). Modal analysis was performed in a finite-element (FE) model to study the oscillation response in isolated thin corneal flaps/eye globes and to analyse the dependency of the frequency response function on: corneal elasticity, viscoelasticity, geometry (thickness and curvature), IOP and density. The model was verified experimentally in flaps from three bovine corneas and in two enucleated porcine eyes using sound excitation (100–110 dB) together with a phase-sensitive OCT to measure the frequency response function (range 50–510 Hz). Simulations showed that corneal vibration in flaps is sensitive to both, geometrical and biomechanical parameters, whereas in whole globes it is primarily sensitive to corneal biomechanical parameters only. Calculations based on the natural frequency shift revealed that flaps of the posterior cornea were 0.8 times less stiff than flaps from the anterior cornea and cross-linked corneas were 1.6 times stiffer than virgin corneas. Sensitivity analysis showed that natural vibration frequencies of whole globes were nearly independent from corneal thickness and IOP within the physiological range. OCT vibrography is a promising non-invasive technique to measure corneal elasticity without biases from corneal thickness and IOP.

## 1. Introduction

The transparent part of the outer eye coat—the cornea—is the main refractive component of the eye. The inherent microstructure defines the material properties of the cornea, which in turn determine its shape. Deviations from the optimal aspheric corneal shape produce optical degradation of the retinal image leading to a decrease in visual function. Therefore, measurement of corneal biomechanical parameters is essential for the characterization of the cornea in certain pathologies that compromise corneal structure (such as keratoconus), as well as prior to and following treatments, such as after refractive surgeries (LASer Insitu Keratomileusis, PhotoRefractive Keratectomy, Intrastromal Corneal Ring Segment implantation), UV collagen cross-linking treatment and keratoplasty. Measuring corneal biomechanical properties allows a better prediction of surgical outcomes, helps to improve the understanding of collagen cross-linking and could permit the early diagnosis of keratoconus. A current limitation in measuring corneal biomechanics in clinics is that very few systems are applicable *in vivo*. Two of the commercially available systems (Ocular Response Analyzer [1] and Corvis ST [2]) analyse the geometrical corneal deformation following an air-puff. This measure however is dependent on several side factors, such as intraocular pressure (IOP), the presence of the sclera and the dynamics of the ocular humours. Ultrasound techniques [3] estimate corneal elasticity by analysing the shear wave, but are limited by a relatively low imaging resolution and the need of direct contact to the eye, which reduces patient comfort. Another promising technique addressing the *in vivo* measurement of quasi-static (GHz vibration) corneal biomechanical properties is Brillouin microscopy [4].

Both, the dynamic and static properties of the cornea are of interest. Viscoelasticity describes a time-dependent material property, which can only be assessed in a dynamic measurement. Ideally, the viscoelastic properties could be used to predict the temporal evolution of the corneal shape under a given IOP and be indicative of a pathologic cornea.

In mechanical engineering, modal analysis [5] is typically applied to study the dynamic behaviour of a material [6]. Thereby the natural frequencies are identified by exciting the sample mechanically at different frequencies and by recording the oscillation amplitude at given locations. The measured frequency response function depends on the inherent mechanical and geometrical properties of the material. In a recent study, a novel imaging approach has been presented to measure the function of the eardrum by imaging the modal shapes occurring during vibration [7]. A phase-triggered optical coherence tomography (OCT) device technique termed OCT vibrography was used to capture the motion, while the eardrum—which is a natural thin membrane with well-defined resonance modes—was exposed to sound emitted from a loudspeaker.

We have recently shown that this technique can induce vibrations in the eye globe and that different harmonics can be observed in the range from 50 to 350 Hz [8].

Here, we describe model-based numerical calculations of natural vibration frequencies for the corneal tissue in flaps and whole eyes and provide supporting experimental results. We use the model to predict the corneal response to vibration in healthy and cross-linked tissue and verify the results with the experimental data obtained from OCT vibrography. Furthermore, we simulate local stiffness alterations to predict the potential corneal response of pathologic tissue. This work demonstrates the possibility of retrieving corneal biomechanical properties from OCT measurements.

## 2. Material and methods

A numerical model was built in order to describe the vibration response of the cornea. Experiments were performed using a custom-developed high-speed OCT system synchronized with a loudspeaker. The experimentally measured frequency response functions were taken as input to fit the biomechanical parameters of the model in order to match the simulated vibration response. The retrieved set of parameters was used in a sensitivity analysis to study the factors that determine the natural frequencies. The effect of corneal elasticity on the natural vibration frequency response was studied in simulations and experiments on corneal flaps from the anterior and posterior cornea (as these regions are expected to show different elasticity) and in virgin and cross-linked corneas (as the cross-linking treatment is expected to stiffen the cornea).

### 2.1. Finite-element simulations

Resonance occurs if a system is excited by one of its natural frequencies which depend on the interaction of the material's elasticity and mass. Modal analysis was performed using proprietary software (ANSYS APDL 14.0—Academic; ANSYS, Inc., Canonsburg, PA, USA). This analysis allows studying the vibration characteristics, i.e. natural frequencies and mode shapes of a structure.

#### 2.1.1. Theory

Generally, motion of a structural system is described by the following equation:2.1
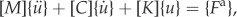
where [*M*] is the structural mass matrix, [*C*] is the structural damping matrix, [*K*] is the structural stiffness matrix, {*ü*} is the nodal acceleration vector, 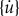 is the nodal velocity vector, {*u*} the nodal displacement vector and {*F*^a^} the applied load vector. We assumed that the external stimulation by the sound wave imposes all nodes in the structure to move at the same frequency. Thereby, the presence of damping and viscoelasticity induces phase shifts, which make the natural frequencies and shapes complex. In this context, equation (2.1) can be expressed by2.2
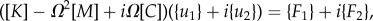
where *u*_1_ and *u*_2_ are the real and imaginary displacements, *F*_1_ and *F*_2_ are the real and the imaginary forces, *i* is the square root of −1 and *Ω* is the imposed circular frequency expressed in radians/time (2*π*f), with the imposed frequency *f* expressed in cycles/time.

In structural dynamic analysis, loads are applied at much higher rates than the natural frequency of the system, which implies that inertia forces and damping need to be considered. Inertia is proportional to the square of the imposed frequency *Ω* and depends on the element mass matrix, whereas damping is directly proportional to the imposed frequency and depends on the element damping matrix. The effects of static or quasi-static loads (such as the IOP) depend on the element stiffness matrix only and are independent of the imposed frequency.

A full solution method was applied in order to directly solve equation (2.2) using a sparse solver (included in the ANSYS code). The imposed frequencies (50–750 Hz) were selected in a slightly wider range than the experimentally scanned frequencies (50–510 Hz). As viscoelastic damping increases the width of the resonance peaks, the frequency spacing was tested between 1 and 50 Hz to find the minimally necessary but least computationally demanding frequency step size to detect the natural frequencies.

#### 2.1.2. Geometry and boundaries

Two different axisymmetric FE models were built to simulate (i) corneal flaps and (ii) whole eye globes.

The flap geometry was simulated by a rectangular cross section of the circular corneal flap (diameter = 4 mm, thickness = 120 µm) and consisted of 54 solid structural elements (2D 4-node, PLANE182). The fixation in the flap holder was implemented by constraining all nodal displacements at the external border to zero. An initial stress of 0.67 kPa was applied to the corneal tissue in order to simulate the minor stress induced by the circular fixation.

The eye globe geometry was simulated by the cross section of a half eye globe, including cornea, limbus, sclera and aqueous humour consisting of 80, 12, 182 and 1234 structural solid elements (2D 8-node, PLANE183), respectively. Table 1 shows the main geometrical and material parameters of the model. It was assumed that the anterior and posterior cornea have a different elasticity, as suggested by Hennighausen *et al*. [9]. Geometries of the cornea, limbus and sclera were set to values taken from the literature [10]. Thereby, the deformations occurring due to pre-stressing the tissue with the IOP were considered by defining a homogeneously distributed initial stress equivalent to the IOP (2.0–2.4 kPa) in all ocular tissues. The ocular geometry in the relaxed eye (i.e. before IOP application) was defined according to table 1. The interior of the eye coat was defined as a fluid, representing the aqueous and vitreous humours, that was modelled by two-dimensional axisymmetric harmonic acoustic elements (FLUID29). Thereby, fluid structure interactions (FSIs) were considered up to a distance of 2.8 mm from the ocular tissues. In order to prevent global motion, the sclera was constrained in a circular ring of 1 mm width at a distance of 5 mm from the apex, simulating the fixation by the eye holder.

**Table 1.** Geometrical and biomechanical parameters used for the FE simulations.

cornea	unit	flap	globe
central thickness	µm	120	865
density	kg m^−3^	1160	1160
Poission's ratio	—	0.499	0.499
Stiffness ant/post cornea	—	1	1.2 (virgin) 2.4 (cxl)
relative modulus	—	0.1	0.1
relaxation time	ms	1	1
anterior curvature	mm	—	6.8
posterior curvature	mm	—	5.57
corneal diameter	mm	4.00	10.40

limbus	unit	globe	
stiffness	kPa	37.2	
Poisson's ratio	—	0.499	
density	kg m^−3^	1160	

sclera	unit	globe	
sclera diameter	mm	19	
stiffness	kPa	79.2	
Poisson's ratio	—	0.499	
density	kg m^−3^	1160	

humours	unit	globe	
density	kg m^−3^	1000	
sonic velocity	m s^−^	1480	
viscosity	kg (s · m)^−1^	8.94 × 10^−4^	

#### 2.1.3. Material model and biomechanical properties

Owing to the nature of modal analysis, material nonlinearities were not considered. Although the cornea is prone to have nonlinear properties, the small experimental deformations observed during sound excitation, i.e. 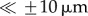 vertical displacement—indicative of much lower values of longitudinal strain—allow the assumption of a linear elastic material model in these simulations. Corneal elasticity was left as a variable in order to adjust the modal response to the experimentally measured frequency response function. Geometrical and biomechanical parameters for limbus and sclera were defined according to table 1.

The temporal nonlinearity of the corneal tissue was implemented by using a viscoelastic material model which was described by a two-parameter Prony series (*p*_1_ = 0.1/*T*_1_ = 1 ms). The relaxation time *T*_1_ was chosen in the range of the sound frequencies and the relative modulus was set within the range of the experimentally observed peak width values.

An acoustic fluid material model was applied to describe the aqueous and vitreous humours. For fluid elements that were closer than 2.8 mm to the ocular structures (cornea, limbus and sclera), FSIs were considered. Fluid material properties were set similar to water, as shown in table 1.

The modal analysis allowed retrieving the natural frequencies and modal shapes for corneal flaps and whole eye globes under different conditions.

### 2.2. Experiments

The value of corneal elasticity used in the simulation was first adjusted in order to match the experimentally measured natural frequencies and then used to evaluate differences in the frequency response function.

#### 2.2.1. Phase-sensitive optical coherence tomographic

A phase-sensitive OCT system was used in the measurements. The instrument is an updated version of the system presented by Chang *et al.* in 2012 [11]. Briefly, the frequency domain OCT is based on a wavelength swept source (*λ* = 1280 nm, Δ*λ* = 125 nm) with an output power of 40 mW. The system was operated at an A-line rate of 48 kHz and a sensitivity of 100 dB. The system is coupled to a high-fidelity loudspeaker driven by sinusoidal signals from a function generator. A multifunctional board was used to acquire the interference data, to control a two-axis galvanometer beam scanner and to trigger the signal of the function generator, allowing synchronization of the data acquisition, beam scanning and sample oscillation. The lateral scanning velocity was dependent on the sampling rate of points per oscillation period at one location.

#### 2.2.2. Experimental protocol

Measurements were performed for a frequency range of the loudspeaker from 240 to 520 Hz or 655 Hz (one case) with frequency steps of Δ*f* = 40 Hz (in flaps) and from 50 to 510 Hz with Δ*f* = 20 Hz (in eye globes). The frequency ranges were selected according to estimations where the natural frequencies might be found. In whole eyes, for each frequency a cross section of the central cornea (diameter 6–8 mm) was imaged at a sound pressure level between 100 and 110 dB. As a reference—in order to eliminate potential motions of the entire set-up—a rigid glass mounted next to the cornea was included at the border of each A-scan. In flaps, the central zone with a diameter of 3–4 mm was imaged. Here, the sound pressure level was lower, which made the glass reference unnecessary in this condition.

#### 2.2.3. Eyes/conditions

**Figure 1.**
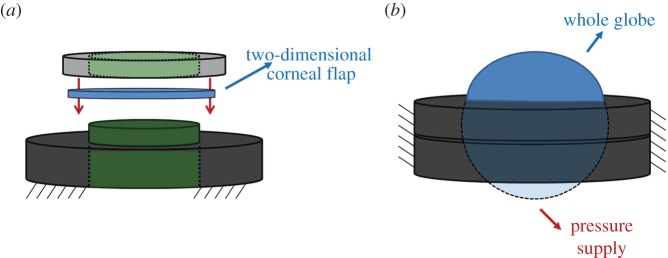
Holders used for the different boundary conditions: (*a*) for two-dimensional corneal flaps, where green shaded zones indicate a cavity in the holder; (*b*) for whole eye globes, where blue shaded zones indicate interiorly pressurized volumes.

A total of five freshly enucleated eyes (two porcine and three bovine) were used within 1–12 h post-mortem. Different boundary conditions for the cornea were tested: (i) two-dimensional corneal flap mounted in a custom holder and (ii) eye globe mounted in a customized holder (figure 1). Flaps of about 120 µm thickness were obtained with a custom-built microkeratome (Deriva, Valencia, Spain) from the anterior and posterior region of the cornea. Eye globes were prepared and measured in three consecutive steps: (I) virgin eye without any treatment; (II) after de-epithelialization and instillation of 0.125% Riboflavin-10% Dextran for 30 min and (III) after irradiation with UV-light (370 nm, 3 mW cm^−2^) for 30 min. Performing step II and III is equivalent to the standard so-called Dresden protocol for UV collagen cross linking, which increases corneal elasticity, with the only difference that the Dextran content of the photosensitizer solution has been reduced from 20% to 10% in order to prevent excessive corneal dehydration. This set of different boundary and treatment conditions allowed studying the effect of geometry, corneal elasticity and changes due to dehydration.

#### 2.2.4. Data processing

The vertical (transversal) oscillation of the central cornea was analysed for each frequency and condition: firstly, the oscillation of the glass reference was subtracted from the overall oscillation measured at the cornea. Then the resulting signal was Fourier transformed in order to extract the exact amplitude at the frequency of interest. This step was necessary as the loudspeaker did not emit a totally single-frequency signal, but contained contributions of higher harmonics at certain frequencies. In order to obtain the frequency response curve, the amplitude of the central corneal oscillation was plotted as a function of frequency.

### 2.3. Analysis

The corneal oscillation amplitude at the apex as a function of frequency was compared for simulation and experiment and the elastic modulus was iteratively adjusted until both sets matched. In a subsequent step, the corresponding mode shapes were compared to ensure that the same vibration mode was present at the resonance peaks. For the best-fit parameter set then a sensitivity analysis was performed to study the dependency on different geometrical (thickness and curvature) and biomechanical (corneal elasticity and density) parameters, as well as on other side factors (IOP, presence of sclera). Although the model has the potential to predict the effect of viscoelasticity, the experimental data would have had to be recorded with a much smaller step-size in order to allow the actual retrieval of viscoelastic parameters.

## 3. Results

### 3.1. Corneal flaps

The experimentally measured frequency response function and mode shapes of flap vibration were best predicted with a corneal elasticity of *E*_flap_ = 14.58 kPa in the model. Using the sampling rate of the experiments, shifts of the resonance frequency peaks could only be registered if they were larger than 40 Hz. This induced an estimated uncertainty of 4.37 kPa (30%) in the retrieved corneal elasticity.

**Figure 2.**
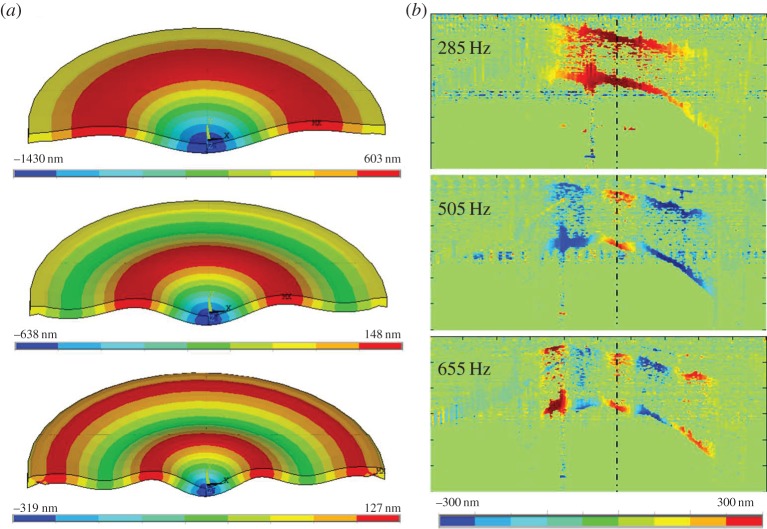
(*a*) Experimentally measured mode shapes at different frequencies in a posterior bovine flap. (*b*) Corresponding harmonics of the fundamental mode in corneal flaps, predicted from simulations in the range from 220 to 750 Hz.

The numerical simulation predicted three different mode shapes (harmonics of the fundamental mode) within the range from 50 to 750 Hz, as shown figure 2*a*. Figure 2*b* illustrates the different mode shapes observed experimentally in a posterior flap. While figure 2*a* shows the 180° axis-symmetric expansion of the model with deformations scaled by factor 50 (for a better perception of the mode shapes), figure 2*b* shows the cross section of the experimental flap with real scale deformations (symmetry lines are plotted for better orientation).

**Figure 3.**
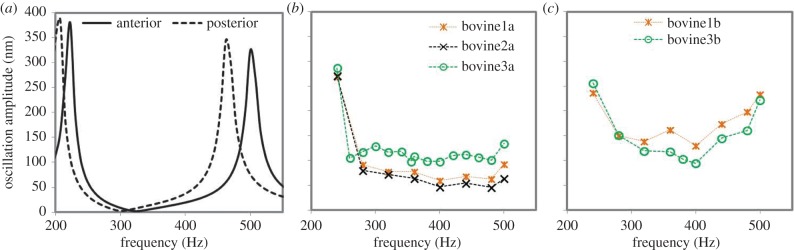
Frequency response function of the (*a*) simulated anterior and posterior corneal flaps, experimentally measured (*b*) anterior flaps and (*c*) posterior bovine flaps. (Online version in colour.)

Figure 3*a* shows the predicted frequency response functions for corneal flaps. Simulations reproduced well the experimental frequency response of anterior bovine flaps as shown in figure 3*b*. Posterior flaps, as in figure 3*c*, showed a second resonance peak in the tested range, whereas anterior corneal flaps only showed one full resonance peak and the beginning of the second peak. Optimal correspondence between the simulated and the experimental posterior cornea was obtained when the flap elasticity was decreased by a factor of 0.8 with respect to the anterior flap (from 14.58 kPa to 11.6 kPa). Each peak in figure 3*a* corresponds to a different mode shape. This indicates that the first two modes in figure 3 are presented near resonance, while the third mode is the beginning of its formation.

**Figure 4.**
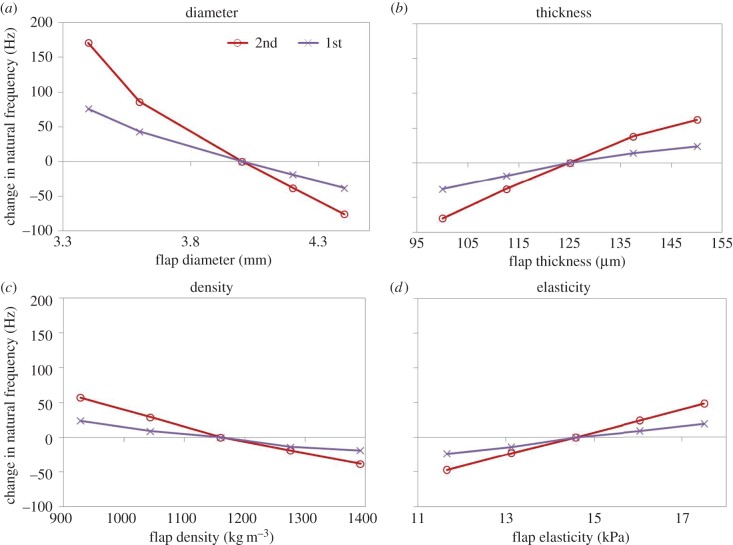
Predicted dependence of the change in first and second natural frequencies with (*a*) flap diameter, (*b*) flap thickness, (*c*) flap density and (*d*) flap elasticity, from a sensitivity analysis. (Online version in colour.)

For the sensitivity analysis, the relative contribution of different parameters on the natural frequencies and their shift were analysed. Figure 4 shows the observed dependencies of the resonance frequency on the different parameters, for a parameter variation of ±20%. Table 2 shows the corresponding slopes of linear regressions to the curves in figure 4. This analysis reveals that the resonance frequency was most sensitive to the geometry of the membrane (its radius, followed by its thickness) and to a lesser extent to density and corneal elasticity. Thereby, the second resonance peak was more sensitive to changes in corneal elasticity than the first peak. As a general tendency, a shift of the resonance peaks to higher frequencies also increased the distance between resonance peaks.

**Table 2.** Sensitivity gradient of geometrical and biomechanical parameters with respect to different resonance peaks, analysed in a range of ±20%.

flap		first	second			unit
stiffness	14.58 kPa	7.48	16.26			Hz Pa^−1^
density	1160 kg m^−3^	−93.95	−205.14			Hz mg^−1^ km^−3^
thickness	125 µm	1256.00	2880.00			Hz mm^−1^
radius	4.0 mm	−111.69	−237.73			Hz mm^−1^

eye globe		first	second	third	fourth	unit
density	1214 kg m^−3^	0.00	−24.71	−137.56	−183.69	Hz mg^−1^ km^−3^
stiffness	24.8 kPa	1.19	3.23	6.73	7.18	Hz Pa^−1^

### 3.2. Whole eye globes

The experimentally measured frequency response function and mode shapes of whole globe vibration were best predicted with a corneal elasticity of 24.8 and 19.8 kPa for the anterior and posterior virgin cornea, respectively.

**Figure 5.**
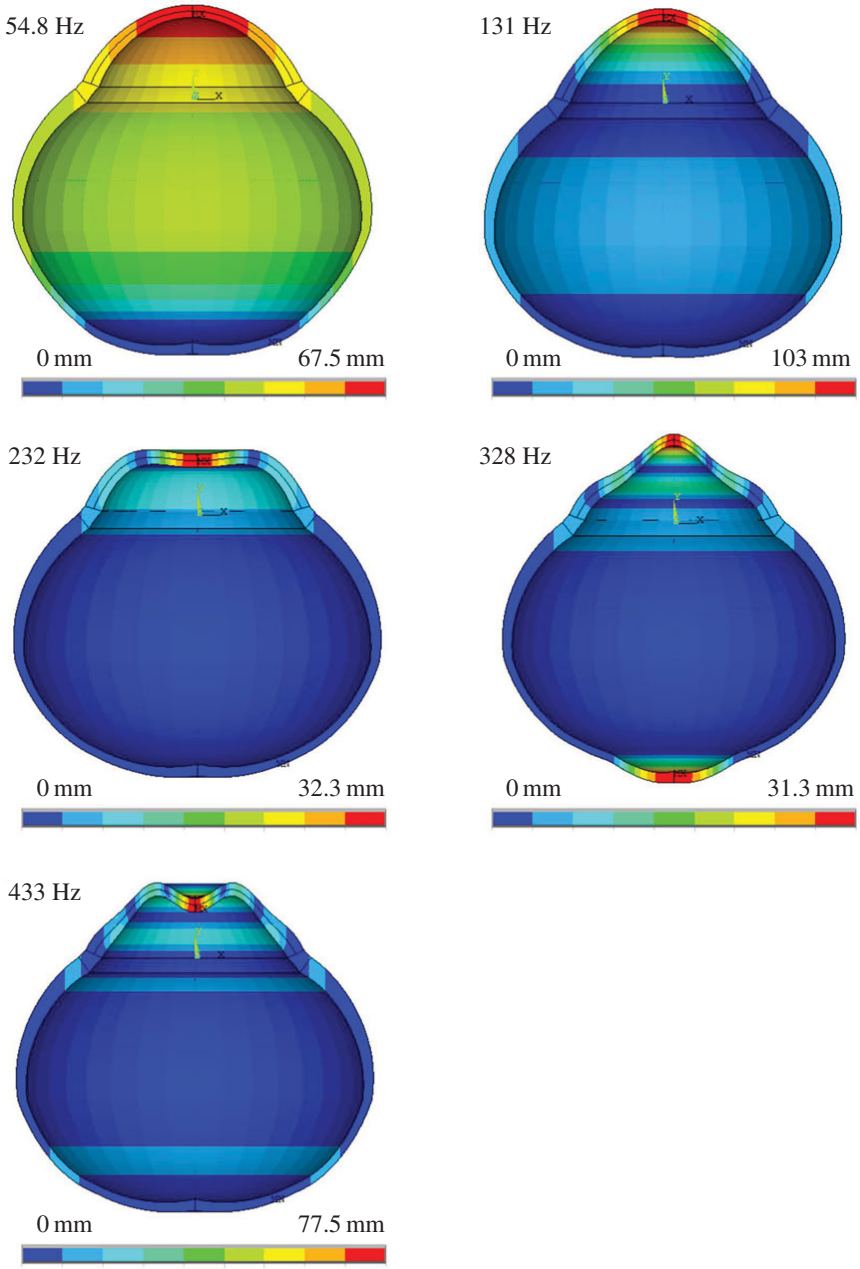
Harmonics of the fundamental mode in whole globes, predicted from simulation in the range from 50 to 510 Hz.

Simulations predicted five different mode shapes (symmetric modes only) in whole eyes within the studied frequency range from 50 to 510 Hz (figure 5). Thereby, modes with only minor scleral deformations are most interesting in the retrieval of corneal mechanical parameters.

**Figure 6.**
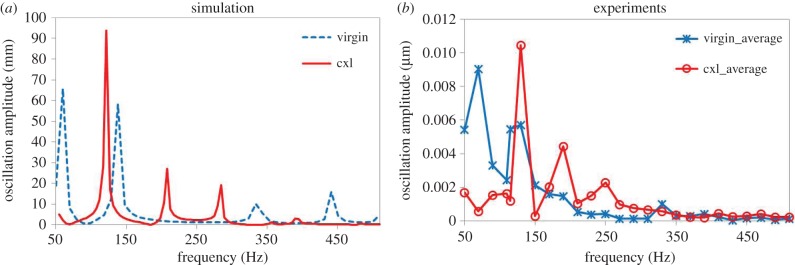
Frequency response function of the (*a*) simulated and (*b*) experimental (average of two eyes per condition) whole globes, virgin (in blue) and cross linked (in red).

Figure 6 compares the simulated frequency response functions for virgin and cross-linked corneas. An increase in corneal elasticity by a factor of 1.64 (from 24.8 to 40.6 kPa) predicted well the experimentally observed changes in the frequency response function after cross linking. The experimental amplitudes are generally lower than in the simulations. The simulated modal analysis presents the maximal possible oscillations, but in practice the amount of deformation depends on the sound pressure level of the sound wave, the energy coupling from sound to tissue and the general damping of the sound wave within the material. Therefore, in the current analysis only the natural frequencies and the mode shapes were analysed, but not the actual values of the oscillation amplitude.

**Figure 7.**
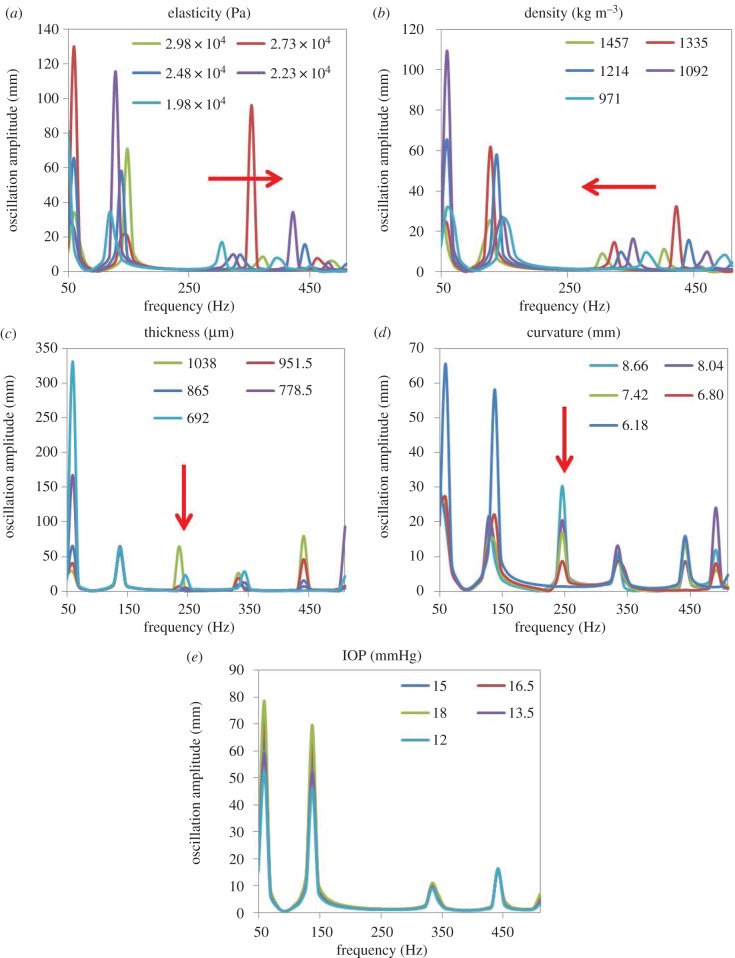
Simulated frequency response functions of whole eye globes for different (*a*) corneal elasticity, (*b*) density, (*c*) thickness, (*d*) curvature and (*c*) IOP values. Red arrows indicate changes in the frequency response function when increasing the corresponding parameter.

**Figure 8.**
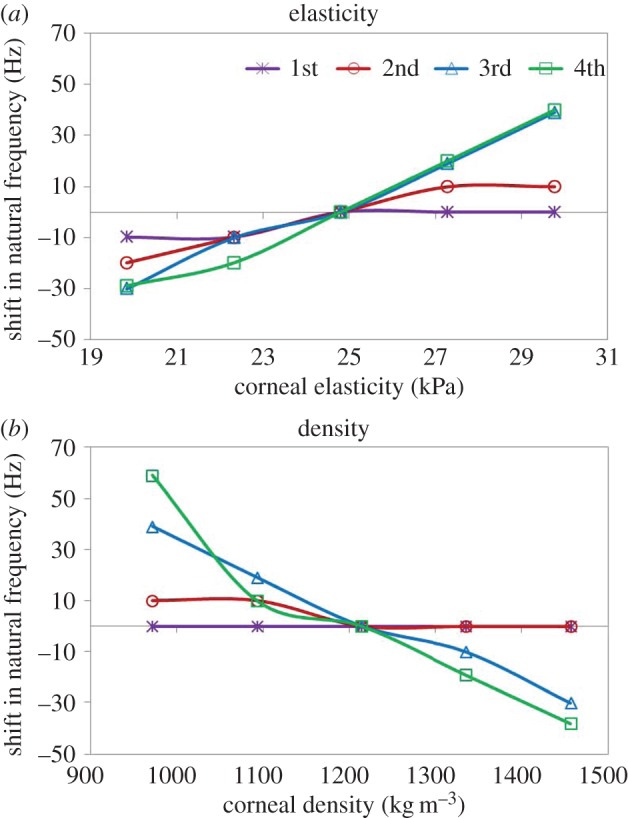
Predicted dependency of the natural frequency (first–fourth) shifts on (*a*) corneal elasticity and (*b*) corneal density. (Online version in colour.)

Figure 7 addresses the results of the sensitivity analysis: individual dependencies of the natural frequencies of a whole eye globe under different parameter variations (by ±20%) are shown. Thereby, changes in corneal elasticity and density induced a shift in the natural frequencies showing a strong, linear dependency (figure 8). Table 2 summarizes the corresponding slopes of the linear trend. Corneal thickness and curvature played a role in the presence of the natural frequency at 250 Hz, whereas the IOP had no significant effect.

**Figure 9.**
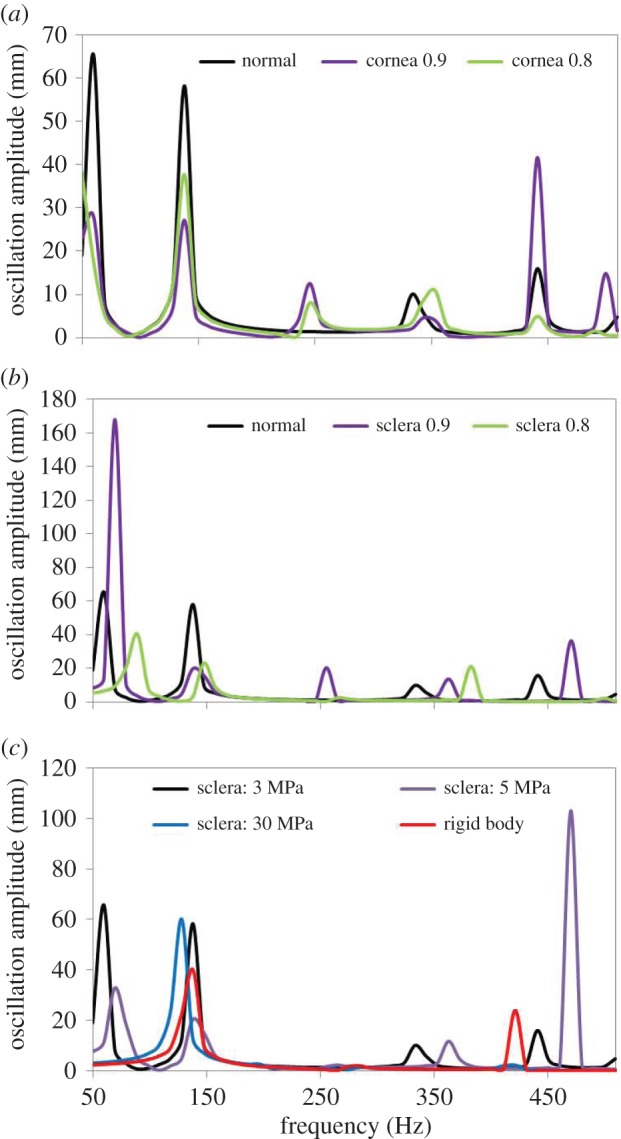
Predicted shifts in the frequency response function of the whole globe upon variations in (*a*) diameter of the cornea, (*b*) diameter of the sclera and (*c*) biomechanical properties of the sclera. The legend in (*a*,*b*) indicates the factor by which the dimensions of the cornea and sclera are modified. The legend in (*c*) indicates the scleral elasticity.

Figure 9*a*,*b* shows the simulated frequency response function of a whole globe when corneal and sclera dimensions were changed independently which allowed us to evaluate the role of the geometry of each eye component. We observed that changes in corneal diameter induced predominantly changes in the resonance amplitude, while changes in the sclera dimension induced a shift of the natural frequency. The effect of the biomechanical properties of the sclera on the frequency response function of whole eye globes was further explored by simulating different sclera elasticity values, i.e. low (74.4 kPa), normal (744 kPa) and an infinite (rigid body). Figure 9*c* shows that sclera biomechanical properties played a role in the presence of the resonance peak at 59 Hz and induced a frequency shift, particularly at higher resonance frequencies.

**Figure 10.**
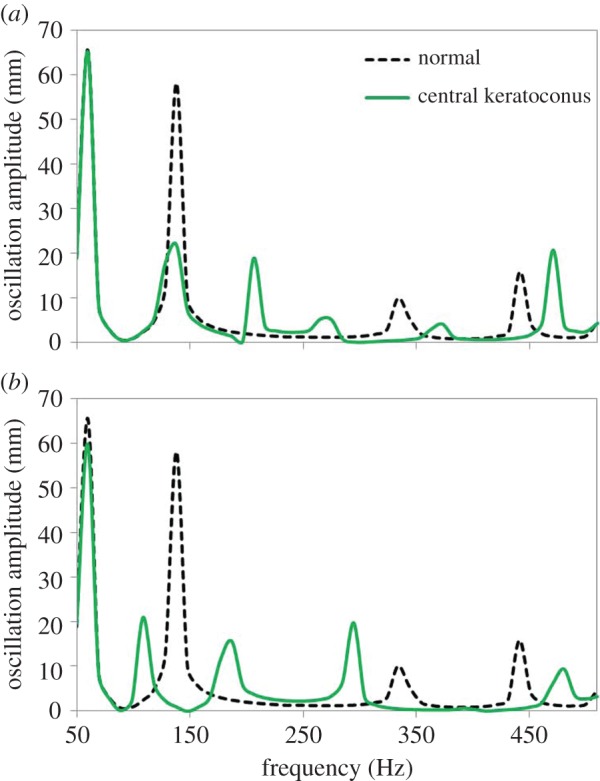
Predicted frequency response function in whole eye globes, for a normal cornea (dashed line) and for corneas with a localized reduction of corneal elasticity (solid line), with the weakened area located (*a*) centrally (70°–90°) or (*b*) in a peripheral ring (50°–70°). (Online version in colour.)

Figure 10 shows changes in the frequency response function for a simulated simple keratoconic eye. Figure 10*a* presents a central keratoconus, simulated by reducing corneal elasticity in an angular zone from 70° to 90°, and figure 10*b* presents an idealized keratoconic cornea where the corneal elasticity has been reduced in a peripheral ring from 50° to 70°. Angles are defined from 0°, at the scleral equator, to 90°, at the corneal apex, considering the axis symmetry of the model. Corneal elasticity was reduced in the selected area from 24.8 to 2.48 kPa for the simulation. In both cases, changes in the frequency response function were predicted: in the central keratoconus, new resonance peaks appeared at 206 and 275 Hz, and frequency shifts occurred for the resonances at 334 and 441 Hz, whereas in the peripheral keratoconus a new resonance peak appeared at 187 Hz and frequency shifts occurred for the resonances at 138, 334 and 441 Hz. In contrast to figures 2 and 5 where the mechanical parameters were changed homogeneously along the cornea, local variations in corneal elasticity in the simulations of keratoconic corneas resulted in different trends for the individual resonance modes, both in the direction of frequency shift and in the difference in amplitude across frequencies.

## 4. Discussion

We present a numerical model to study the natural frequencies of the cornea under vibration, from which corneal biomechanical properties of flaps and whole eyes were obtained. We furthermore provide supporting experimental data from a new non-contact OCT-based vibrography technique. The advantage of OCT vibrography is that the resonance-based approach is more directly related to the inherent mechanical properties of the corneal tissue than methods relying on measuring the corneal deformation following an air-puff [2,12,13] or the water volume needed to provoke a certain increase in the IOP [14].

In standard clinical approaches (Goldmann applanation and air-puff tonometers), the interactions between corneal deformation, thickness and biomechanical properties require the application of specific correction formula [15] to assess the real IOP. However a vibrography-based approach had been previously proposed for acoustic tonometry, where corneal thickness variations were predicted to only have a minor effect on the shift of resonance frequencies (12% thickness variation leads to a 2% frequency shift) [16]. Our simulation results confirm that natural frequencies of the eye are practically independent from its thickness within the physiologic range.

The use of OCT vibrography as a tonometer has been proposed as an alternative to Goldmann applanation approaches [17]. Simulations [18] predicted a moderate dependency on the IOP (Δ = 0.42–1.8 Hz mmHg^−1^), especially for higher resonance frequencies. We performed a subset of simulations with a sampling rate of 1 Hz and could confirm a very minor dependency on IOP (Δ = 0–0.14 Hz mmHg^−1^). A clinical study [19] revealed that the instrument was in fact rather insensitive to IOP variations and therefore not appropriate for the use in tonometry. Although that was a negative result for the attempted use of the instrument as a tonometer, the independence of the frequency response on IOP—as also demonstrated by our simulations—is actually a very positive result for the use of this technology to assess the biomechanical properties of the cornea. Also, comparing the IOP gradient to the elasticity and density gradients in table 2 shows that biomechanical properties seem to be much stronger correlated to a shift in the resonance frequencies than IOP. Hence, the current vibrography technique potentially allows the estimation of corneal biomechanics without being significantly affected by side factors. The small sensitivity to thickness and IOP is a strong advantage over previous techniques designed to study the corneal biomechanics, such as the ocular response analyser (Reichert, Depew, NY, USA) and air-puff corneal deformation imaging—such as the Corvis Scheimpflug system (Oculus, Wetzlar, Germany)—or non-contact tonometers, where special correction formulae need to be applied in order to exclude the effect of thickness in the IOP measurement.

This advantage arises as vibrography uses a small-strain approach, whereas macroscopic deformation techniques such as in air-puff or indentation tonometery use a large-strain approach. Biomechanical properties in a small stress–strain range can be considered more sensitive to the corneal microstructure and hence are more likely to be observed in the physiologic/pathologic conditions than macroscopic deformations, which would rather happen during trauma.

Although the IOP was not related to resonance frequency shifts, we observed a strong relation between the oscillation amplitude of the cornea and the actual IOP, which agrees with the observation of von Freyberg *et al*. [20]. Corneal OCT vibrography therefore can be envisioned as a two-in-one diagnostic tool to assess (i) corneal elasticity by analysis of the resonance frequency and (ii) IOP by analysis of the oscillation amplitude after subtracting the contribution of thickness, curvature and corneal elasticity.

The spatial shapes of the resonance modes in figure 8 indicate that the deformation occurs primarily at the cornea, suggesting that the resonance modes are more sensitive to corneal than to scleral biomechanical properties. This was further confirmed by the sensitivity analysis, where a 40% change in sclera elasticity accounts for a frequency shift of 25 Hz, while the same amount of change in corneal elasticity accounts for a frequency shift of 49 Hz.

In flap simulations we observed the trend that the viscoelastic parameters were related to the width of the resonance peaks and to the oscillation amplitude. However, the experimental step size was too large to retrieve the real width of the resonance peaks and hence this effect could not be further investigated.

As shown in figure 10, the frequency response function is also sensitive to local differences across the cornea. Weakening in a certain location will favour or hinder the formation of one mode or another. Although our two-dimensional model only allowed us to study axis-symmetric corneal weakening, the results suggest that OCT vibrography might also be useful in the diagnosis of keratoconus, as the oscillation pattern is affected by the location of the cone and the amount of local corneal elasticity decrease. As clinically, the cone is typically placed non-centrically, a three-dimensional model will be required for more detailed analysis.

Corneal elasticity estimated from the simulations agrees with previous literature in the way that the posterior corneal elasticity is less stiff than the anterior cornea (factor 1.2 versus 1.16) [9] and that cross-linked corneas are stiffer than virgin corneas (factor 1.6 versus 1.3 to 1.6) [21]. Absolute corneal elasticity values obtained from OCT vibrography (14.6–24.8 kPa) were similar to values obtained from magnetic resonance imaging (40–185 kPa) [22] and ultrasound (190 kPa) [23], but much smaller than from strip-extensiometry (1.30–11.1 MPa) [24]. This difference might be explained by considering the speed of the measurement technique: stress–strain extensiometry can be considered a static testing approach, where vibrography (50–500 Hz), ultrasound (50 MHz) and magnetic resonance imaging (300 MHz) are dynamic approaches.

We found minor differences between the retrieved corneal elasticity of flaps and whole eyes. Possible reasons for the discrepancy include the fact that the flap is pre-stressed upon mounting in the holder and that the corneal mechanical properties in the entire cornea vary along its thickness. Besides, the predictions indicate that a finer experimental frequency sampling is desirable to guarantee that all resonance peaks are captured within the scanned area. A further experimental limitation was that corneal hydration could not be controlled during the measurements. However, the freshness of the tissue and the constancy of the specimen thickness during the measurements suggest that hydration changes were not an issue.

Theoretically, the phase lag between stress (periodic application of force, i.e. sound) and strain (subsequent deformation) is associated with the material's viscoelastic behaviour. However, as the time delay between the function generator signal and the emission of the sound wave was unknown, the experimental phase lag could only be estimated but did not show sufficient consistency to provide reliable information regarding the dynamic modulus, i.e. the ratio between the storage and loss modulus. Some early literature already proposed the use of the phase lag between viscous and elastic force as vibration tonometer, as it was assumed to depend on the applied stress (i.e. IOP) [25,26]. However, the technique did not make its way into clinics, probably due to the significant biomechanical differences between patients.

Another measured parameter—which simulation predicted to be indicative for viscoelastic properties, at least in corneal flaps—is the width of the resonance peak. The simulations show that increased sound frequency sampling can be used to obtain also viscoelastic parameters.

The oscillation amplitudes observed experimentally reach up to 300 nm (flaps)/10 µm (whole eye). These are larger than the oscillations induced by ultrasound, but orders of magnitude smaller than the corneal indentation induced by an air-puff tonometers, and hence very non-invasive to the corneal tissue. In corneal vibrography, the oscillation amplitude depends on the actual stress–strain interactions within the corneal tissue, allowing a more direct estimation of the corneal mechanical properties. Ultrasound methods rely on the assumption that the corneal acoustic impedance is correlated with corneal elasticity, which has been demonstrated for low strain levels only [23,27]. Similarly, Brillouin microscopy relies on the relationship between the Brillouin and Young's modulus, which has been proven experimentally against rheology measurements [28].

Further experimental developments for OCT vibrography should include comprehensive *ex vivo* evaluations in mounted whole eyes under different treatments and improvements towards *in vivo* configuration, such as the use of a focused sound wave exciting the cornea to avoid the discomfort of high sound volume during measurements and a lower corneal laser irradiance. An additional potential effect on the frequency response function *in vivo* (*in situ*) may arise from the ocular muscles and surrounding fatty tissue, which will probably lead to an additional resonance peak but not further affect the corneal resonances. This factor can be addressed computationally by modelling of ocular muscle damping and experimentally by comparing *in vivo* with *ex vivo* samples. A further improvement of the model would be the extension to three dimensions, which will allow studying the effect of asymmetry, such as fibril orientation or in pathologies like keratoconus.
